# Supplementary material for: Long-range skin Josephson supercurrent across a van der Waals ferromagnet
Source: Nat Commun. 2023 Mar 30;14:1779. doi: 10.1038/s41467-023-37603-9 (PMC10063542; doi:10.1038/s41467-023-37603-9)
Supplement: Supplementary file 1 — Supplementary Information [file 41467_2023_37603_MOESM1_ESM.pdf]

Long-range skin Josephson supercurrent across a van der Waals ferromagnet

Guojing Hu[1#], Changlong Wang[1#], Shasha Wang[1], Ying Zhang[1], Yan Feng[1], Zhi Wang[2*], Qian Niu[3], Zhenyu Zhang[4], Bin Xiang[1*]

[1]*Department of Materials Science & Engineering, CAS Key Lab of Materials for Energy Conversion, Anhui Laboratory of Advanced Photon Science and Technology, University of Science and Technology of China, Hefei 230026, China*

[2]*School of Physics, Sun Yat-sen University, Guangzhou 510275, China*

[3]*School of Physical Sciences, University of Science and Technology of China, Hefei 230026, China*

[4]*International Center for Quantum Design of Functional Materials (ICQD), University of Science and Technology of China, Hefei 230026, China*

Supplementary Note 1: Ferromagnetism of Fe_3GeTe_2 (FGT)

Fig. 1b shows the crystal structure of FGT. It can be seen that FGT is composed of layered Fe/FeGe/Fe, sandwiched between two Te atom layers. The Fe_3Ge substructure contains two kinds of Fe atoms: Fe I and Fe II. The Fe I sites are fully occupied, and the Fe II sites are partially occupied. Therefore, Fe II atoms easily form vacancies during sample growth. The Fe_3GeTe_2 single crystals were grown by a chemical vapor transport (CVT) method, and the magnetic properties were measured by SQUID. The *M-T* curve of bulk FGT shows a clear ferromagnetic-paramagnetic transition at around 200 K (Supplementary Fig. 8a). The *M-B* curve of bulk FGT indicates that the bulk FGT has a strong perpendicular magnetic anisotropy with an easy magnetization direction along the c-axis (Supplementary Fig. 8b) and shows almost the same value of coercive field (~ 250 Oe) under the *in-plane* and the *out-of-plane* magnetic fields (inset of Supplementary Fig. 8b). When the thickness of the FGT flake decreases, the flake coercive field increases and the thinner the FGT flake is, the closer the ratio of remnant magnetization to saturation magnetization (M_R/M_S) is to 1, which shows the hard magnetic property[1]. The anomalous Hall resistance of the FGT flake with a thickness of 10 nm shows that the coercive field of the FGT flake under the *out-of-plane* magnetic field is 0.38 T, which is far less than that of 1.18 T under the *in-plane* magnetic field (Supplementary Fig. 20). Many researchers have observed that FGT has a topological chiral magnetic structure through different measurement methods. For example, Wang et al. reported angular dependence of the topological Hall effect (THE) in the uniaxial FGT, generated by either the possible topological domain structure or the noncoplanar spin structure forming during the in-plane magnetization[2]. Kim et al. utilized theoretical calculations, magnetotransport, and angle-resolved photoemission spectroscopy to reveal that FGT is a topological nodal line semimetal[3], where the orbital-driven nodal line can be tunable by spin orientation and can produce a large Berry curvature, which leads to a large anomalous Hall current. Some researchers directly observed magnetic skyrmions and chiral spin textures[4-6] by using

nanoscale magnetic imaging techniques, such as Lorentz transmission electron microscopy and scanning electron microscopy with polarization analysis.

Supplementary Note 2: The decay length ξ of the supercurrent

Due to the exchange-field-driven pair breaking, the spin-unpolarized singlet supercurrents in a ferromagnet will decay rapidly. Hence, the coherence length of the superconducting current $\xi_{\mathrm{singlet}}^{\mathrm{FM}}$ is typically very short, on the order of a nanometer or less[7-9]. $\xi_{\mathrm{singlet}}^{\mathrm{FM}} \approx \sqrt{\frac{\hbar D}{2E_{\mathrm{ex}}}}$, where D is the electron diffusion coefficient, and E_{ex} is the exchange energy in the ferromagnet FGT[10]. $D = \frac{\hbar^2(3\pi^2)^{2/3}}{3m_n e^2 n^{1/3}\rho}$, where m_{n} is the effective electron mass, which can be approximated as the mass of free electrons $m_0 = 9.1 \times 10^{-31} kg$. Here, e is the electron's charge $e = 1.6 \times 10^{-19} C$, and n is the electron carrier density. The electron carrier density of FGT can be obtained by the Hall effect. The calculation process is as follows:

(1) First, we need to get the Hall resistivity through the measured Hall resistance (Supplementary Fig. 11d) $\rho_{\mathrm{xy}} = R_{\mathrm{xy}} * d$, where d is the thickness of the FGT. Then the relationship curve of the Hall resistivity with the applied magnetic field (ρ_{xy}-H) was plotted.

(2) The Hall resistivity has a linear relationship with the external magnetic field H, with $\rho_{\mathrm{xy}} = R_{\mathrm{H}} * H$, and its slope can be obtained by a linear fitting, which is the Hall coefficient R_{H}.

(3) The electron carrier density can be obtained from the Hall coefficient, $n = 1/e * R_{\mathrm{H}}$.

Finally, we calculate the carrier density as $2.5 \times 10^{28}\, m^{-3}$. Letting ρ denote the resistivity of FGT, $\rho_{\mathrm{xx}} = \frac{R_{xx}*w*d}{L} = 1.27 \times 10^{-7} \Omega \cdot m$. $E_{ex} \cong 2\pi k_{\mathrm{B}} T_{\mathrm{Curie}}$, where k_{B} is the Boltzmann constant, and T_{Curie} is the Curie temperature of FGT (200 K). Therefore, the $\xi_{\mathrm{singlet}}^{\mathrm{FM}}$ of FGT is calculated to be 3.5 nm, which is much smaller than

the channel length in the $NbSe_2$/FGT/$NbSe_2$ lateral Josephson junction. However, many studies show that the spin-polarized triplet supercurrents can prevail over very long length scales[11-13], scales much longer than expected for singlet correlations. Assuming that spin-flip scattering and spin-orbit scattering are not considered, spin-triplet supercurrents are limited by a thermal coherence length, $\xi_{\mathrm{triplet}}^{\mathrm{FM}} \approx \sqrt{\frac{\hbar D}{2\pi k_{\mathrm{B}} T}}$. When the temperature is 3 K, the $\xi_{\mathrm{triplet}}^{\mathrm{FM}}$ can be calculated as 40.7 nm.

Experimentally, when only the channel length of the device is different, using an exponential decay function to fit the superconducting critical current I_c versus barrier length L_j data, can estimate a decay length of Josephson supercurrents. However, the different devices in our experiments have different channel length L_j, width, and thickness, which result in different cross-section areas. As a result, it may not be an appropriate way to draw the L_j-dependent superconducting critical current I_c. It is a challenge for us to fabricate identical devices with all the same dimension parameters except the different channel length by this mechanical exfoliation and dry transfer method. There is an alternative way to demonstrate the L_j-dependent behavior of the Josephson junctions: The characteristic voltage I_cR_n product as an important parameter of Josephson junction has been reported to demonstrate the strength of the Josephson coupling without the sample-specific geometrical factors and can be utilized to estimate a decay length of Josephson supercurrents[14-17]. Therefore, in our case, the characteristic voltage I_cR_n product can be employed to estimate our junction decay length. The mean-free path l in Fe_3GeTe_2 layer is significantly shorter than the coherence length ξ and we can take our Josephson junction into "dirty limit" $l < \xi$.[18,19] As a result, to qualitatively demonstrate the decay tendency in the Fe_3GeTe_2 spacer, we fit the L_j-dependent characteristic voltage I_cR_n product using an exponential decay function, $\exp(-\frac{L_j}{\xi})$, which is appropriate in the "dirty limit" junction regime to estimate the decay length of Josephson supercurrents through the Fe_3GeTe_2 barrier approximately[19-22]. As shown in the inset of Fig. 2c, the estimated ξ = 227±18 nm at 3 K under zero magnetic field is

much longer than the theoretically calculated coherence length, but comparable to the channel length of our device.

Supplementary Note 3: Superconductivity of $NbSe_2$

$NbSe_2$ has a layered hexagonal structure, where the Nb atom is located in the center of the triangular Se prism. The magnetic measurement of the $NbSe_2$ single crystal was carried out by SQUID. When the temperature drops below the critical temperature of $NbSe_2$, a negative magnetic susceptibility appears, indicating that $NbSe_2$ has complete diamagnetism below the transition temperature (Meissner effect). The *M-T* curve shows that the superconducting temperature of the $NbSe_2$ single crystal is 7 K (Supplementary Fig. 1a). The *M-B* curve in Supplementary Fig. 1b indicates that $NbSe_2$ is a Type II superconductor with lower critical magnetic field B_{c1} and upper critical magnetic field B_{c2}. The critical magnetic field, critical temperature, and critical current are the three parameters of superconductivity. Supplementary Fig. 1c shows the temperature-dependent resistance of exfoliated thin layers of $NbSe_2$ under different vertical magnetic fields. When the temperature drops to around 6.9 K, the resistance suddenly drops to zero, signifying the critical temperature. Supplementary Fig. 1d shows the temperature-dependent resistance of exfoliated thin layers of $NbSe_2$ under different in-plane magnetic fields. Applying an external magnetic field can destroy the superconductivity. With the increase of the applied magnetic field, the superconducting transition temperature of $NbSe_2$ becomes lower. When the applied magnetic field exceeds a certain value, the superconductivity of $NbSe_2$ is completely destroyed, and this magnetic field is called the critical magnetic field. It can be seen that $NbSe_2$ has strong anisotropy and is more sensitive to the application of vertical magnetic fields. Supplementary Fig. 1e shows the *I-V* curve at different temperatures. When the applied current reaches 1.81 mA at 3 K, $NbSe_2$ transforms into a normal resistance state, and this current is called the critical current. Supplementary Fig. 1f shows the temperature-dependent critical current extracted from Supplementary Fig. 1e; the critical current decreases monotonically with increasing temperature.

Supplementary Note 4: Fitting of BKT transition

Two transition temperatures appear in the *R*-*T* curve at zero magnetic field (Fig. 1d). The first transition temperature T_{c1} appears at 6.9 K, corresponding to the superconducting transition of $NbSe_2$. The second transition temperature of 5.4 K is derived from the proximity effect-induced superconducting transition in the Josephson junction. As the temperature decreases, the resistance gradually decreases to zero resistance at 3.7 K. The tail of the resistance drop can be fitted by the Halperin-Nelson equation describing the Berezinskii–Kosterlitz–Thouless (BKT) transition[23,24].

$$R = R_0 exp^{\left(-2b\left(\frac{T_{c0-T}}{T-T_{\mathrm{BKT}}}\right)^{\frac{1}{2}}\right)} \qquad (1)$$

The R_0 and *b* in the equation are parameters of the material. The fitting result is shown by the solid red line in Fig. 1d, which indicates that at the BKT transition temperature of 3.7 K, the zero-resistance state is driven by the binding of vortex-antivortex pairs.

Supplementary Note 5: Characteristic oscillations in Josephson junctions

The interference pattern of the Josephson current depends on the summation of the phase-sensitive supercurrents between the superconductors[24-26]. When the supercurrent density distribution is symmetrical,

$$I_{\mathrm{c}}(B) = |\int_{-\infty}^{\infty} J_s(x)\cos\left(\frac{2\pi L_{\mathrm{eff}} Bx}{\Phi_0}\right)dx| \qquad (2)$$

where $J_{\mathrm{s}}(x)$ is the Josephson current density, *x* is the real space coordinate along the junction width, and L_{eff} is the effective junction length along the current direction, which should take into account the magnetic flux focusing from the contacts due to the Meissner effect. Φ_0 is the magnetic flux quantum. From the above formula, we can get that the distribution of different current densities causes different interference patterns of the Josephson current. The uniform supercurrent density distribution produces a single-slit Fraunhofer pattern $|(\sin(\pi L_{\mathrm{j}} BW/\Phi_0))/(\pi L_{\mathrm{j}} BW/\Phi_0)|$, which is characterized by a central peak with a width of $2\Phi_0$ and peak intensity on both sides attenuation with $1/B$. In contrast, when the supercurrent density is predominantly distributed along the edges, a sinusoidal double-slit pattern is generated, where the

width of the central peak shrinks to Φ_0, and the attenuation of the peak intensity on both sides is determined by the width of the edge channel.

Supplementary Note 6: Extraction process of supercurrent density distribution

In our junction shown in Fig. 3a, we assume that the magnetic field is applied along the z-axis. The maximum critical current $I_C(B)$ can be viewed as the magnitude of the Fourier transform of $J_S(y)$, so the $J_S(y)$ can be extracted from the inverse Fourier transform (IFT) of the experimentally measured $I_C(B)$. The extraction procedure for the supercurrent density profile is as follows (Supplementary Fig. 13)[25,27].

At a fixed magnetic field, the total critical current is a phase-sensitive summation of the supercurrent along the *x*-direction. The Fourier transform of the supercurrent density $J_S(x)$ can yield a complex critical current $\xi_C(\beta)$

$$\xi_C(\beta) = \int_{-\infty}^{\infty} dx J_S(x) e^{i\beta x} \qquad (3)$$

where $\beta = \frac{2\pi L_{\text{eff}} B}{\Phi_0}$ is the normalized magnetic field unit.

The critical current $\xi_C(\beta)$ can be divided into two parts: $\xi_C(\beta) = I_E(\beta) + iI_O(\beta)$.

where $I_E(\beta)$ represents the even function, $I_E(\beta) = \int_{-\infty}^{\infty} dx J_E(x) \cos\beta x$, and $I_O(\beta)$ represents the odd function, $I_O(\beta) = \int_{-\infty}^{\infty} dx J_O(x) \sin\beta x$.

If considering the current density only as a symmetrical distribution, the odd function part can be ignored, so equation (1) can be written as $\xi_C(\beta) = I_E(\beta) = \int_{-\infty}^{\infty} dx J_E(x) \cos\beta x$. Since the $J_E(x)$ is real and positive, the $\xi_C(\beta)$ is real, and it alternates between positive and negative values at the zero-crossing position. The experimentally observed maximum critical current is approximately equal to $|\xi_C(\beta)|$, so we can get the exact $\xi_C(\beta)$ by flipping the sign of every other lobe of the observed $I_C(\beta)$.

For accuracy, we do need to consider the small odd part of the current density $I_{\mathrm{O}}(\beta)$, so the experimentally observed maximum critical current can be written as $I_{\mathrm{C}}(\beta) = |\xi_{\mathrm{C}}(\beta)| = \sqrt{I_{\mathrm{E}}^2(\beta) + I_{\mathrm{O}}^2(\beta)}$. The $I_{\mathrm{C}}(\beta)$ is dominated by $I_{\mathrm{E}}(\beta)$ except at the minima points, so $I_{\mathrm{E}}(\beta)$ can be obtained by multiplying $I_{\mathrm{C}}(\beta)$ with a flipping function to change the sign between adjacent lobes. However, when $I_{\mathrm{E}}(\beta)$ is minimal, $I_{\mathrm{O}}(\beta)$ dominates the critical current, which can be approximated by interpolating between the minima of $I_{\mathrm{C}}(\beta)$, and flipping sign between lobes. At last, we can get the current density $J_{\mathrm{S}}(x)$ profile by Fourier transform of the complex $\xi_{\mathrm{C}}(\beta)$.

$$J_{\mathrm{S}}(x) = \left|\frac{1}{2\pi}\int_{-b/2}^{b/2} d\beta\, \xi_{\mathrm{C}}(\beta) e^{i\beta x}\right| \qquad (4)$$

Supplementary Note 7: The Josephson supercurrent in a $NbSe_2/Fe_3GeTe_2/NbSe_2$ vertical geometry

We fabricated the $NbSe_2/Fe_3GeTe_2/NbSe_2$ vertical heterojunctions with different thicknesses of Fe_3GeTe_2, the schematic structure and optical photos are shown in the Supplementary Fig. 18. Supplementary Fig. 18c shows the *I-V* curves of Josephson junctions with different FGT thickness measured at 3 K. When the thickness of FGT is 5 nm, the junction shows an obvious Josephson critical current ~250 μA. When the thickness of the FGT increases to 8 nm, a large non-zero voltage component appears in the *I-V* curve, which indicates that the Josephson supercurrent in a $NbSe_2/Fe_3GeTe_2/NbSe_2$ vertical structure does not have a long-range behavior. Therefore, from above results, we can conclude that in our lateral Josephson junction, probably no long-range supercurrent flows in the bulk of FGT layer, and the surface topological supercurrent dominates the junction transports because of surface spin-orbit coupling effect.

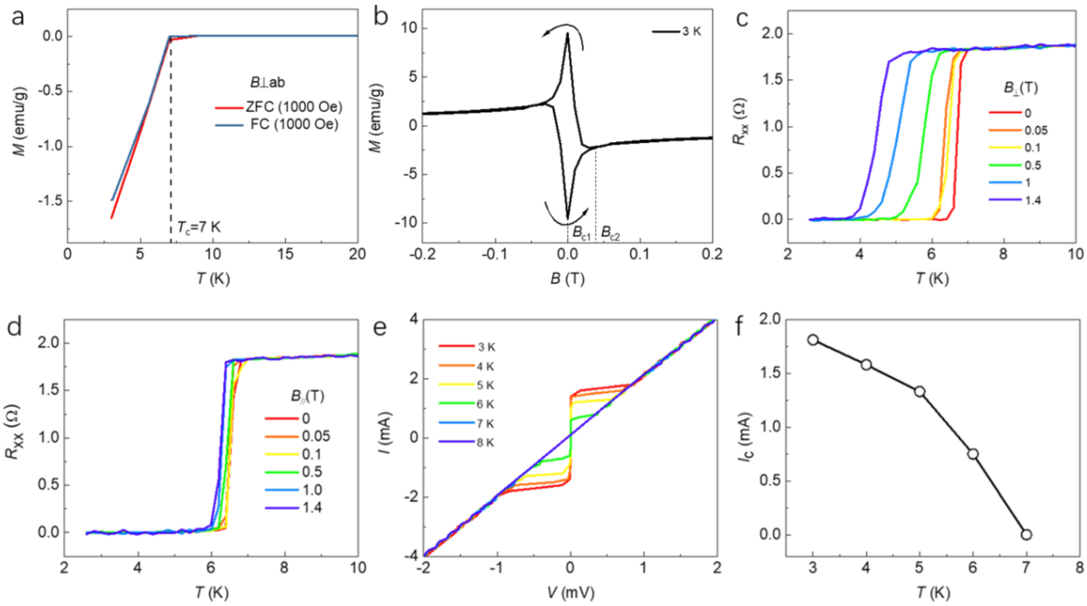

Supplementary Fig. 1. (a) Temperature dependence of the magnetization of single-crystal $NbSe_2$ under different cooling conditions. (b) Magnetization of single-crystal $NbSe_2$ at 3 K as a function of the *out-of-plane* magnetic field. (c) Temperature-dependent resistance curves of few-layer $NbSe_2$ in different *out-of-plane* magnetic fields. (d) Temperature-dependent resistance curves of few-layer $NbSe_2$ in different *in-plane* magnetic fields. (e) Current-voltage curves of few-layer $NbSe_2$ at different temperatures. (f) Critical currents at different temperatures, extracted from (e).

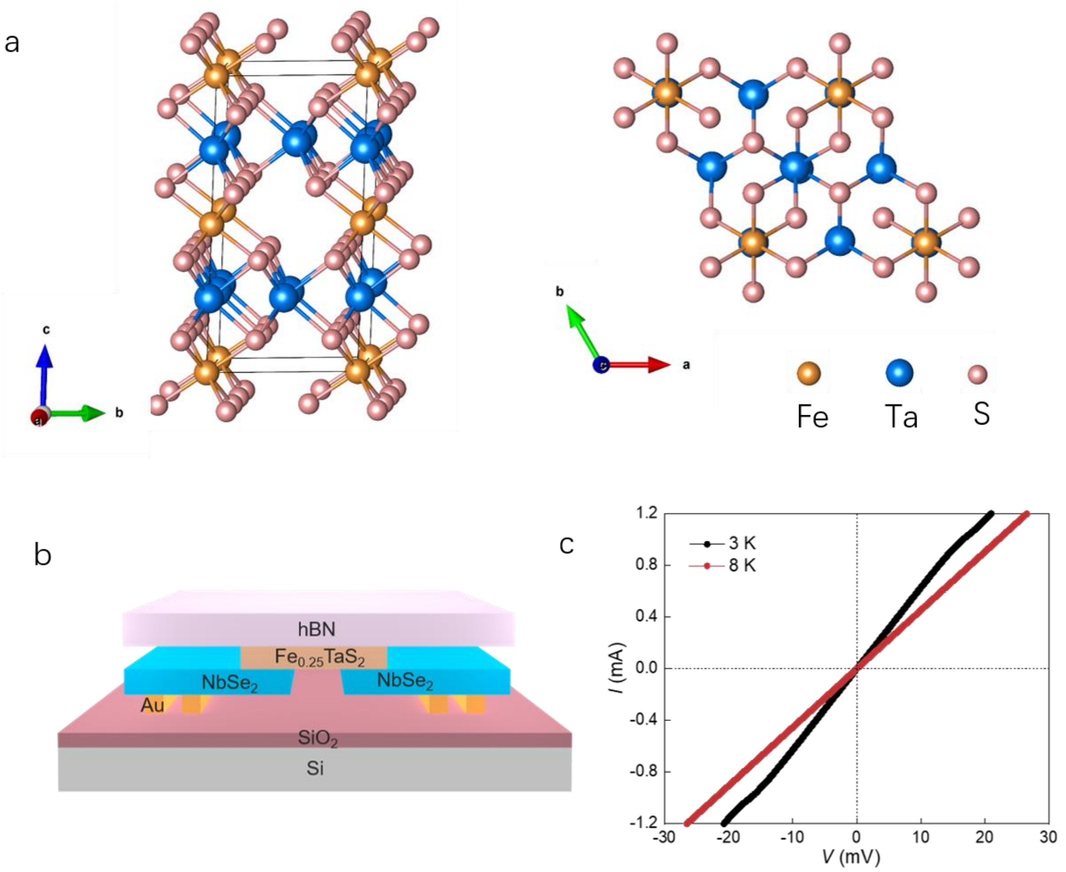

Supplementary Fig. 2. (a) Schematic diagram of the atomic structure of $Fe_{0.25}TaS_2$. (b) Schematic diagram of the $NbSe_2/Fe_{0.25}TaS_2/NbSe_2$ junctions with channel length L_j=200 nm. (c) Current-voltage (*I*-*V*) curves of $NbSe_2/Fe_{0.25}TaS_2/NbSe_2$ junction at different temperatures under zero magnetic field. $Fe_{0.25}TaS_2$ is a ferromagnetic metal with uniform magnetization. There is no supercurrent observed in this $NbSe_2/Fe_{0.25}TaS_2/NbSe_2$ lateral Josephson junction with the channel length of 200 nm.

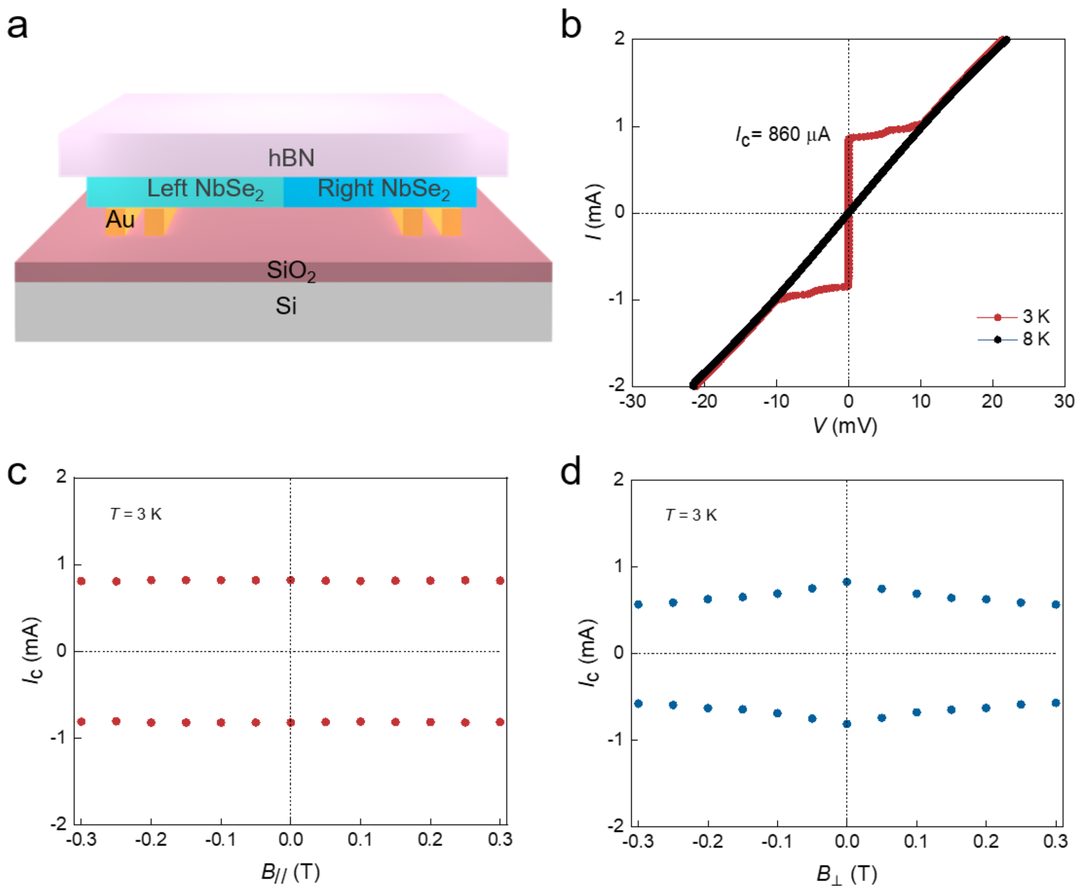

Supplementary Fig. 3. (a) Schematic diagram of the $NbSe_2/NbSe_2$ junctions in short-circuited condition. (b) Current-voltage (*I*-*V*) curves for $NbSe_2/NbSe_2$ junction at different temperatures under zero magnetic field with the junction critical current I_c~860 μA at 3 K. Critical current I_c of $NbSe_2/NbSe_2$ junction as a function of *in-plane* (c) and *out-of-plane* (d) magnetic field at T = 3 K.

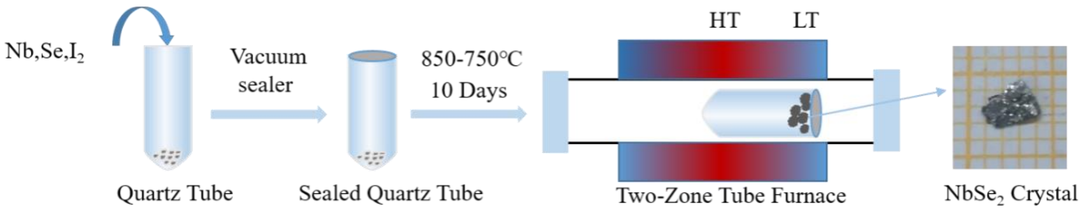

Supplementary Fig. 4. Growth process of $NbSe_2$ single crystal by chemical vapor transport.

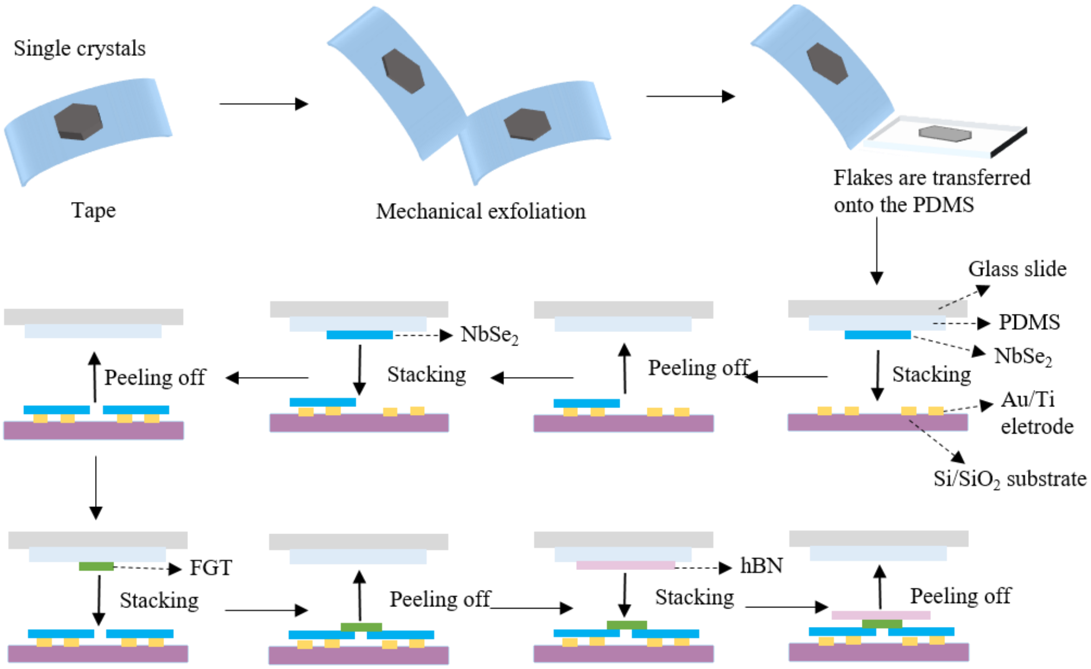

Supplementary Fig. 5. Mechanical exfoliation of single crystals and dry transfer processes of $NbSe_2$/FGT/$NbSe_2$ heterostructures.

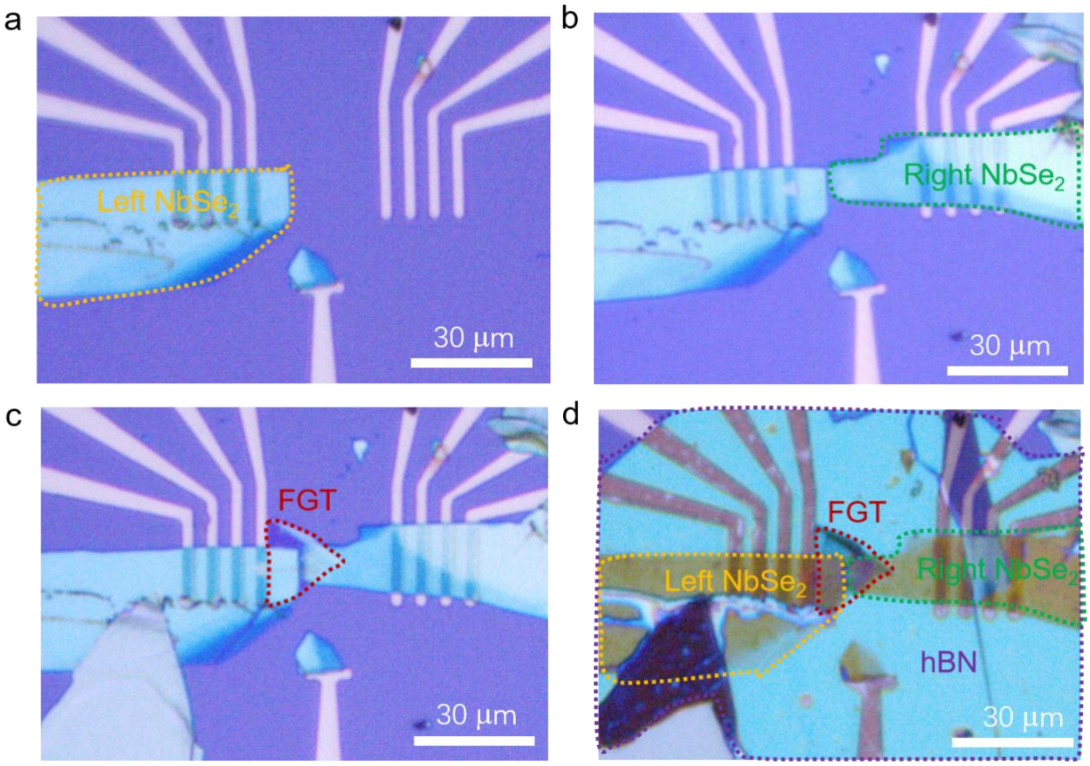

Supplementary Fig. 6. Fabrication process of $NbSe_2$/FGT/$NbSe_2$ lateral Josephson junction device with a channel length of 300 nm. (a) Optical image of the $NbSe_2$ nanoflake transferred onto left side of the parallel electrodes. (b) Another $NbSe_2$ nanoflake stacked onto right side of the parallel electrodes in (a). (c) A layer of FGT was placed on the gap position bridging the two layers of $NbSe_2$. (d) A layer of hBN encapsulating the whole heterostructure in (c) for protection.

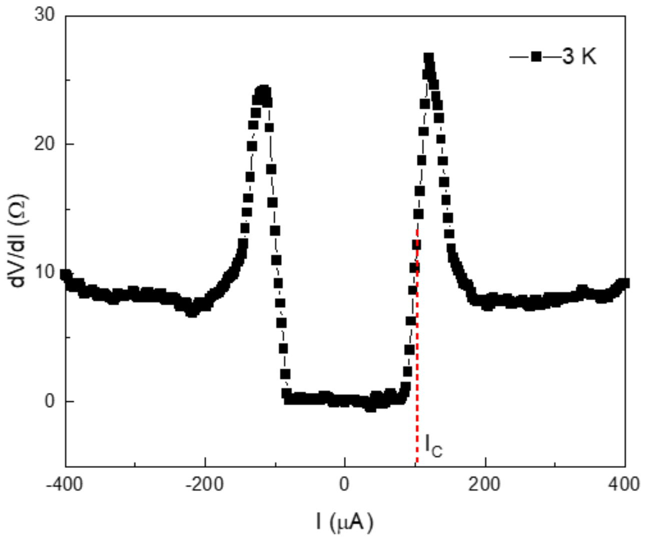

Supplementary Fig. 7. The *I*-d*V*/d*I* curve of Josephson junction at temperatures of 3 K under zero magnetic field, and the position at half of the difference between the maximum value and the minimum value in the *I*-d*V*/d*I* curve is defined as the Josephson critical current I_c.

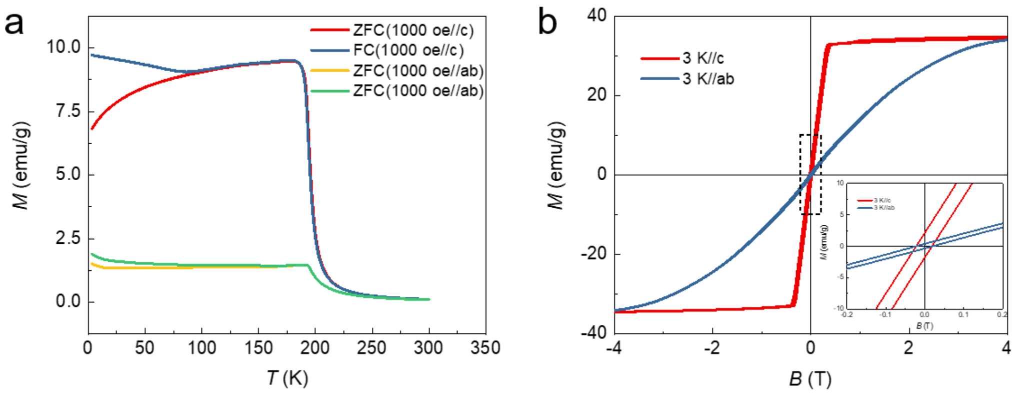

Supplementary Fig. 8. Magnetic properties of bulk Fe_3GeTe_2. (a) Temperature-dependent magnetization curves measured at 1000 Oe external field along both c-axis and ab-plane for as-grown FGT with a Curie temperature of 200 K. (b) Field-dependent magnetization plots of FGT at 3 K. The external field is applied in different directions of *B*//c (red) and *B*//ab (blue), and the inset is the enlarged part in the dashed box.

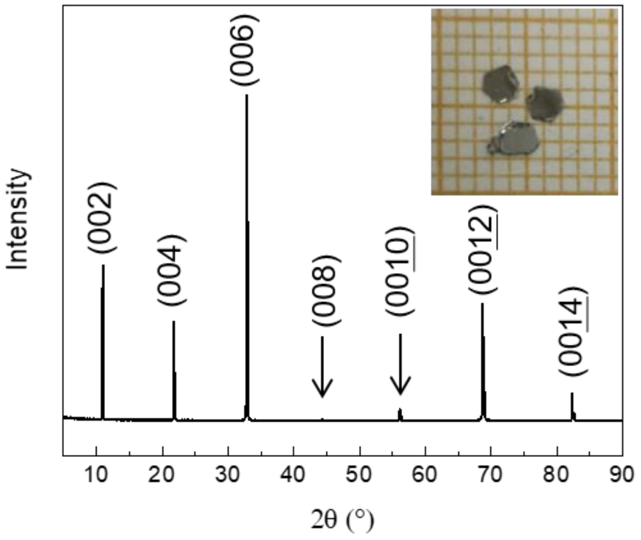

Supplementary Fig. 9. XRD pattern for the as-grown FGT single crystal. The inset is an optical image of the FGT single crystal.

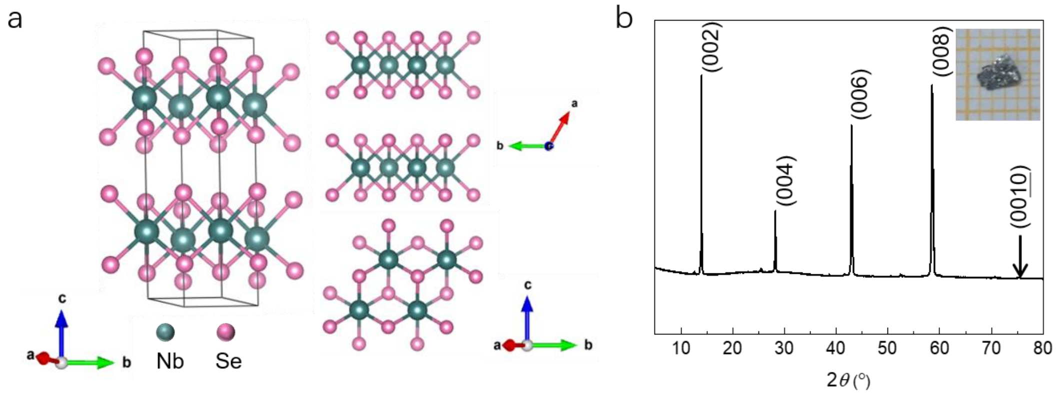

Supplementary Fig. 10. (a) Schematic diagram of the crystal structure of $NbSe_2$ viewed from different directions. (b) XRD pattern of as-grown $NbSe_2$ single crystal. The inset is an optical image of the $NbSe_2$ single crystal.

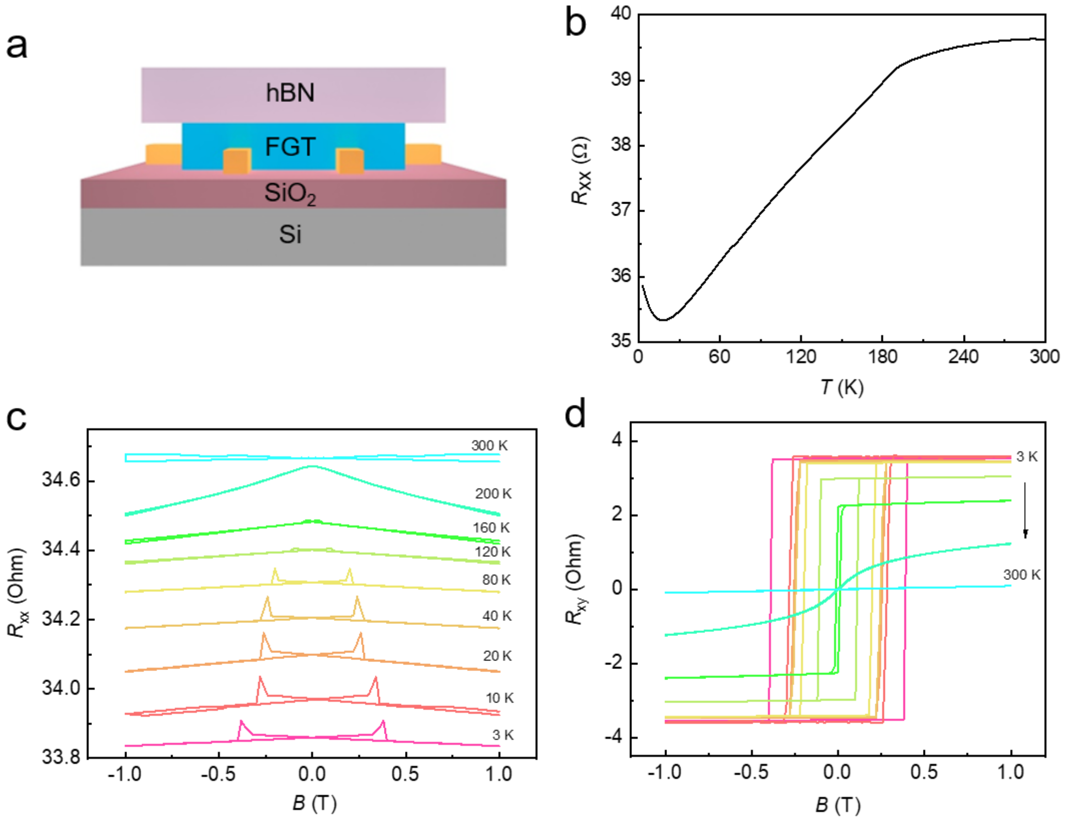

Supplementary Fig. 11. Electrical transport of a few-layer FGT Hall device. (a) Schematic diagram of the FGT Hall device. (b) Temperature dependence of the longitudinal resistance of the FGT Hall device. (c) Magnetic field dependence of longitudinal resistance at different temperatures. (d) Magnetic field dependence of Hall resistance at different temperatures.

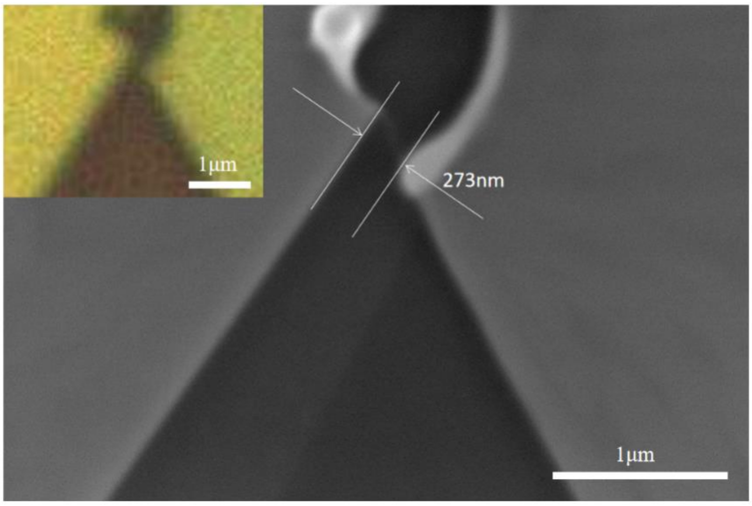

Supplementary Fig. 12. SEM image of cross-sectional areas in $NbSe_2$/FGT/$NbSe_2$ junctions with an inset of the corresponding optical image, proving that the spacing distance of 273 nm measured using SEM is very close to the value of 267 nm measured using optical microscopy.

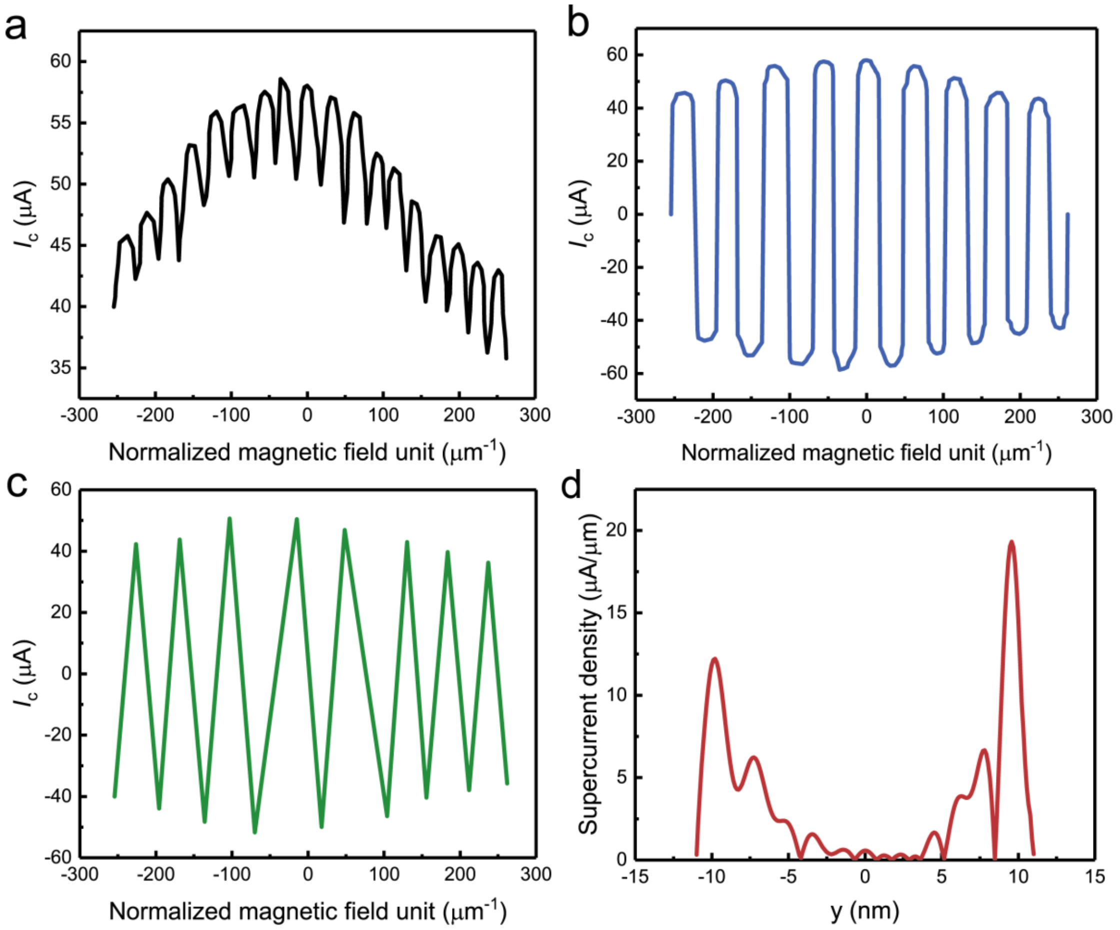

Supplementary Fig. 13. Analysis of superconducting current density. (a) Critical superconducting current as a function of the magnetic field, taken from the differential resistance diagram of Fig. 3b. (b) Recovered critical current $I_E(\beta)$, corresponding to the even-numbered part of the current density distribution J_E(y). (c) Recovered critical current $I_O(\beta)$, corresponding to the odd part J_O(y) of the current density distribution. (d) Distribution of superconducting current density J_S(y).

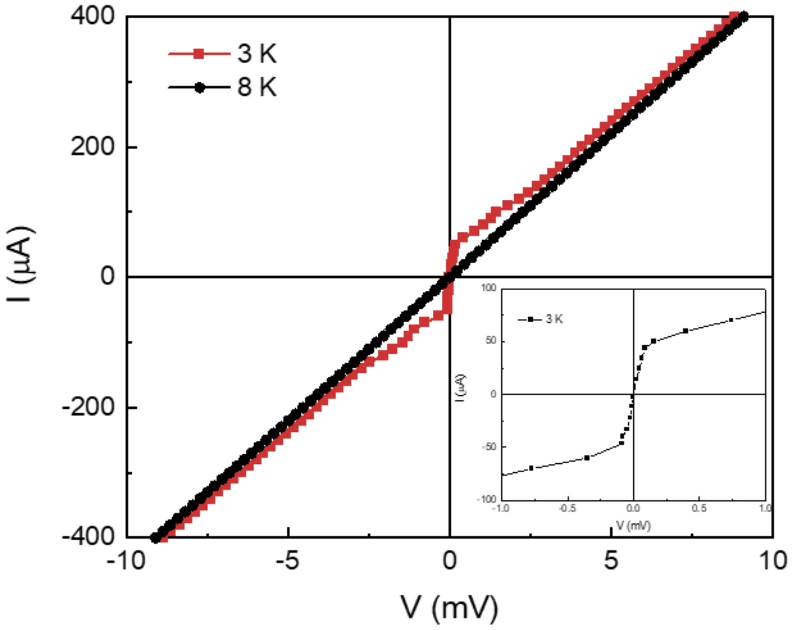

Supplementary Fig. 14. Current-voltage (*I*-*V*) curves of the $NbSe_2$/FGT/$NbSe_2$ junctions with channel length L_j=300 nm at different temperatures under zero magnetic field with the junction critical current I_c~50 μA at 3 K. The inset shows the magnified plots of *I*-*V* curves at temperature of 3 K around zero voltage.

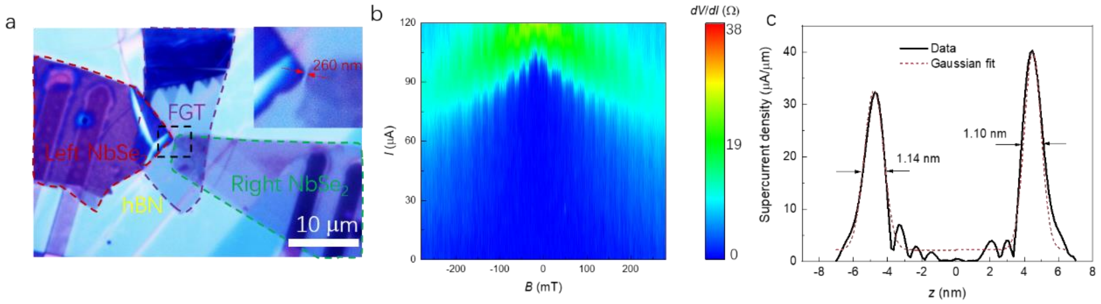

Supplementary Fig. 15. (a) Optical image of the $NbSe_2$/FGT/$NbSe_2$ junctions with channel length L_j=260 nm. The inset in the upper right corner is a magnified view of the junction area framed by the black dashed square. (b) Differential resistance map across the $NbSe_2$/FGT/$NbSe_2$ junction with channel length L_j=260 nm at 3 K with the magnetic field along the *in-plane* direction, showing a double-slit interference pattern. (c), Distribution of supercurrent density along the *y*-axis obtained from the inverse Fourier transform of the data in (b). The width of the surface channel can be estimated by the red dashed line using Gaussian fitting. The FGT layer thickness is 14 nm.

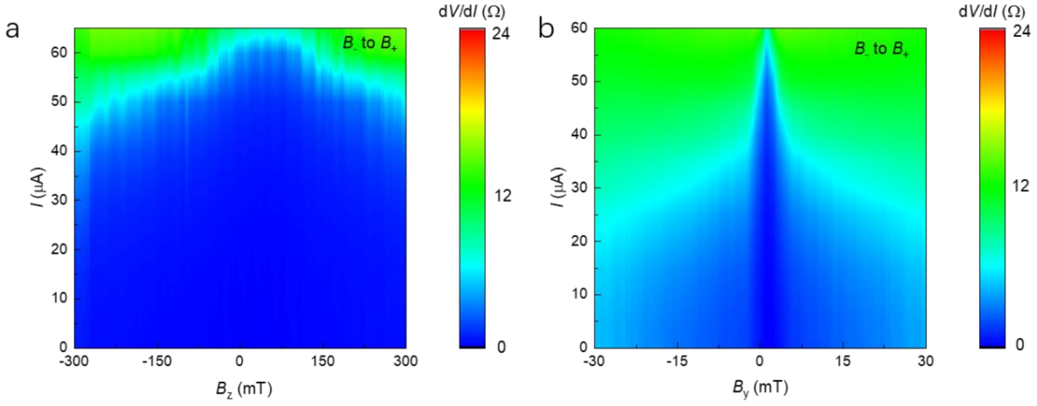

Supplementary Fig. 16. (a) Differential resistance map across the junction at 3 K with the magnetic field scanning from negative to positive, showing a sinusoidal double-slit interference pattern. (b) Differential resistance map across the junction at 3 K with the magnetic field scanning from negative to positive, showing a single-slit interference pattern.

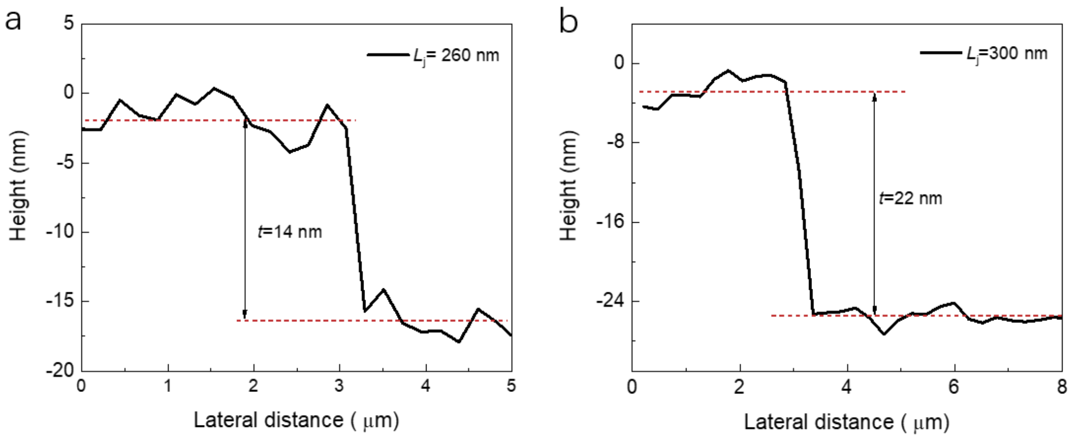

Supplementary Fig. 17. The thickness of FGT in the $NbSe_2$/FGT/$NbSe_2$ junctions measured by atomic force microscope with channel length (a) L_j=260 nm and (b) L_j=300 nm.

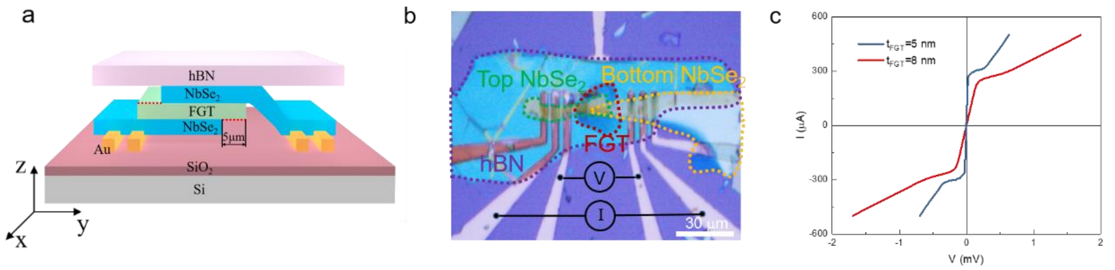

Supplementary Fig. 18 (a) Schematic illustration of $NbSe_2$/FGT/$NbSe_2$ vertical Josephson junction. (b) Optical image of the vertical Josephson junction. (c) Current-voltage (*I*-*V*) curves for vertical Josephson junctions with different FGT thickness at temperatures of 3 K under zero magnetic field.

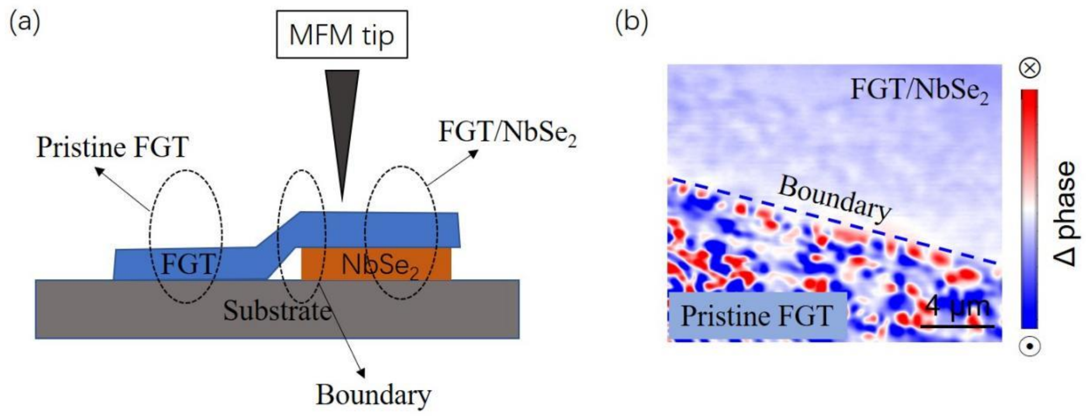

Supplementary Fig. 19 (a) Schematic illustrate of FGT/$NbSe_2$ heterostructure characterized by MFM. (b) Magnetization distribution of the FGT layer across a boundary region of the heterostructure with and without the underlying $NbSe_2$ by MFM at 0 T magnetic field, indicating the superconductivity can weaken the magnetization of the Fe_3GeTe_2 flake.

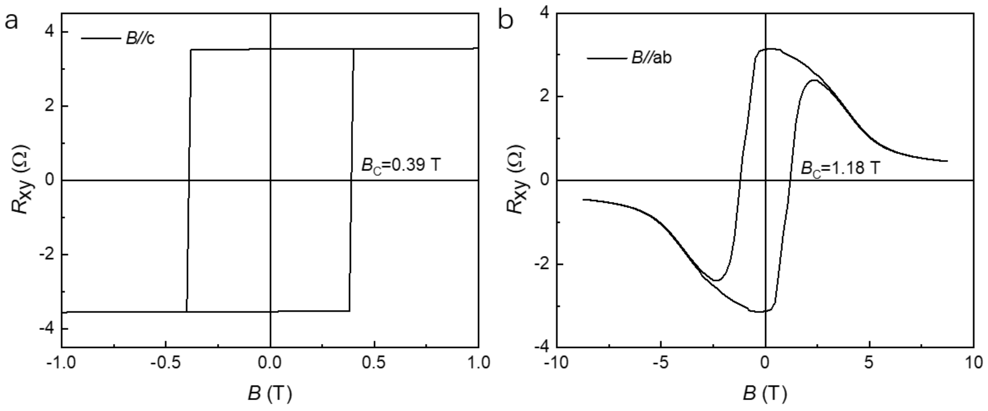

Supplementary Fig. 20 (a) The anomalous Hall resistance of the FGT with thickness of 10 nm under *out-of-plane* magnetic field, and the coercive field B_c is 0.39 T. (b) The anomalous Hall resistance of the FGT with thickness of 10 nm under *in-plane* magnetic field, and the coercive field B_c is 1.18 T.

Supplementary References

1. Tan, C. *et al.* Hard magnetic properties in nanoflake van der Waals Fe_3GeTe_2. *Nat. Commun.* **9**, 1554 (2018).
2. You, Y. *et al.* Angular dependence of the topological Hall effect in the uniaxial van der Waals ferromagnet Fe_3GeTe_2. *Phys. Rev. B* **100**, 134441 (2019).
3. Kim, K. *et al.* Large anomalous Hall current induced by topological nodal lines in a ferromagnetic van der Waals semimetal. *Nat. Mater.* **17**, 794-799 (2018).
4. Meijer, M. J. *et al.* Chiral spin spirals at the surface of the van der Waals ferromagnet Fe_3GeTe_2. *Nano Lett.* **20**, 8563-8568 (2020).
5. Nguyen, G. D. *et al.* Visualization and manipulation of magnetic domains in the quasi-two-dimensional material Fe_3GeTe_2. *Phys. Rev. B* **97**, 014425 (2018).
6. Wang, H. *et al.* Direct observations of chiral spin textures in van der Waals magnet Fe_3GeTe_2. *https://doi.org/10.48550/arXiv.1907.08382*.
7. Bergeret, F. S., Volkov, A. F. & Efetov, K. B. Long-range proximity effects in superconductor-ferromagnet structures. *Phys. Rev. Lett.* **86**, 4096-4099 (2001).
8. Buzdin, A. I. Proximity effects in superconductor-ferromagnet heterostructures. *Rev. Mod. Phys.* **77**, 935-970 (2005).
9. Houzet, M. & Buzdin, A. I. Long range triplet Josephson effect through a ferromagnetic trilayer. *Phys. Rev. B* **76**, 060504(R) (2007).
10. Jeon, K. R. *et al.* Long-range supercurrents through a chiral non-collinear antiferromagnet in lateral Josephson junctions. *Nat. Mater.* **20**, 1358-1363 (2021).
11. Keizer, R. S. *et al.* A spin triplet supercurrent through the half-metallic ferromagnet CrO_2. *Nature* **439**, 825-827 (2006).
12. Buzdin, A. I., Melnikov, A. S. & Pugach, N. G. Domain walls and long-range triplet correlations in SFS Josephson junctions. *Phys. Rev. B* **83**, 144515 (2011).
13. Sanchez-Manzano, D. *et al.* Extremely long-range, high-temperature Josephson coupling across a half-metallic ferromagnet. *Nat. Mater.* **21**, 188-194 (2022).
14. Kontos, T. *et al*. Josephson junction through a thin ferromagnetic layer: negative coupling. *Phys. Rev. Lett*. **89**, 137007 (2002).

15. Kim, M. *et al*. Strong proximity Josephson coupling in vertically stacked $NbSe_2$–graphene–$NbSe_2$ van der Waals junctions. *Nano Lett*. **17**, 6125-6130 (2017).
16. Kang, K. *et al*. Van der Waals π Josephson junctions. *https://doi.org/10.48550/arXiv.2201.09185*.
17. Tinkham, M. *Introduction to Superconductivity* (Dover, 2004).
18. Bozovic, I. *et al*. Giant proximity effect in cuprate superconductors. *Phys. Rev. Lett*. **93**, 157002 (2004).
19. Barone, A. & Paterno, G. *Physics and Applications of the Josephson Effect* 2nd edn (John Wiley & Sons, 1982).
20. Bell, C. *et al*. Proximity and Josephson effects in superconductor/antiferromagnetic Nb/$\gamma-Fe_{50}Mn_{50}$ heterostructures. *Phys. Rev. B* **68**, 144517 (2003).
21. Weides, M., Disch, M., Kohlstedt, H. & Bürgler, D. Observation of Josephson coupling through an interlayer of antiferromagnetically ordered chromium. *Phys. Rev. B* **80**, 064508 (2009).
22. Jeon, K.-R. *et al*. Long-range supercurrents through a chiral non-collinear antiferromagnet in lateral Josephson junctions. *Nat. Mater*. **20**, 1358-1363 (2021).
23. Saito, Y., Kasahara, Y., Ye, J., Iwasa, Y. & Nojima, T. Metallic ground state in an ion-gated two-dimensional superconductor. *Science* 350, 409-413 (2015).
24. Huang, C. *et al.* Proximity-induced surface superconductivity in Dirac semimetal Cd_3As_2. *Nat. Commun.* 10, 2217 (2019).
25. Hart, S. *et al.* Induced superconductivity in the quantum spin Hall edge. *Nat. Phys.* **10**, 638-643 (2014).
26. Pribiag, V. S. *et al.* Edge-mode superconductivity in a two-dimensional topological insulator. *Nat. Nanotechnol.* **10**, 593-597 (2015).
27. Dynes, R. C. & Fulton, T. A. Supercurrent density distribution in Josephson junctions. *Phys. Rev. B* **3**, 3015-3023 (1971).
